# Stopping bDMARDs at the beginning of pregnancy is associated with disease flares and preterm delivery in women with rheumatoid arthritis

**DOI:** 10.3389/fphar.2022.887462

**Published:** 2022-08-03

**Authors:** Maria Chiara Gerardi, Francesca Crisafulli, Antía García-Fernandez, Daniele Lini, Chiara Bazzani, Ilaria Cavazzana, Matteo Filippini, Micaela Fredi, Roberto Gorla, Maria Grazia Lazzaroni, Cecilia Nalli, Marco Taglietti, Andrea Lojacono, Francesca Ramazzotto, Cristina Zanardini, Sonia Zatti, Franco Franceschini, Angela Tincani, Laura Andreoli

**Affiliations:** ^1^ Rheumatology and Clinical Immunology, ASST Spedali Civili; Department of Clinical and Experimental Sciences, University of Brescia, Brescia, Italy; ^2^ Department of Rheumatol, University Hospital Ramón y Cajal, Madrid, Spain; ^3^ Obstetrics and Gynaecology Unit, ASST Garda Ospedale of Desenzano, Desenzano del Garda, Italy; ^4^ Obstetrics and Gynaecology, ASST Spedali Civili, Brescia, Italy

**Keywords:** rheumatoid arthritis, pregnancy, bDMARDs, TNF inhibitors, disease activity, disease flare, pregnancy outcomes

## Abstract

**Objectives:** Women with Rheumatoid Arthritis (RA) can experience flares during pregnancy that might influence pregnancy outcomes. We aimed at assessing the disease course during pregnancy and identifying risk factors for flares.

**Methods:** Data about prospectively-followed pregnancies in RA were retrospectively collected before conception, during each trimester and in the post-partum period. Clinical characteristics, disease activity (DAS28-CRP3), medication use, and pregnancy outcomes were analysed with regard to disease flares.

**Results:** Among 73 women who had a live birth, 64 (88%) were in remission/low disease activity before conception. During pregnancy, a flare occurred in 27 (37%) patients, mainly during first and second trimester. Flares during pregnancy were associated with the discontinuation of bDMARDs at positive pregnancy test (55% of patients with flare vs*.* 30% of patients with no flare, *p* 0.034, OR 2.857, 95% CI 1.112–8.323) and a previous use of >1 bDMARDs (33% of patients with flare vs*.* 10% of patients with no flare, *p* 0.019, OR 4.1, 95%CI 1.204–13.966). Preterm pregnancies were characterised by higher values of CRP [10 mg/L (5–11) vs*.* 3 mg/L (2.5–5), *p* 0.01] and DAS28-CRP3 [4.2 (1.9–4.5) vs*.* 1.9 (1.7–2.6), *p* 0.01] during the first trimester as compared with pregnancies at term. Preterm delivery was associated with the occurrence of flare during pregnancy (flare 27% vs*.* no-flare 7%, *p* 0.034, OR 4.625, 95%CI 1.027–20.829).

**Conclusion:** Preterm delivery in RA patients was associated with flares during pregnancy. Flares occurred more frequently after the discontinuation of bDMARDs at positive pregnancy test. Women with aggressive RA on treatment with bDMARDs should be considered as candidates for continuing bDMARDs during pregnancy in order to reduce the risk of flare and adverse pregnancy outcomes.

## Introduction

In 1938, Philip Hench described a temporary improvement in Rheumatoid Arthritis (RA) during pregnancy, followed by a post-partum flare (Hench, 1938). Retrospective studies between 1938 and the 1980s, lacking objective measures of disease activity, described improvement in up to 90% of RA women during pregnancy followed by post-partum flares in about 80% (Hazes et al., 2011). In these studies, RA women were not treated with specific RA drugs, maybe occasionally with glucocorticoids.

A systematic review of more recent prospective studies, in which objective indices of disease activity were used, found that 60% of patients with RA improve during pregnancy and 47% relapse after delivery (Jethwa et al., 2019). Only two studies included RA women who were on treatment with conventional disease-modifying anti-rheumatic drugs (cDMARDs) (de Man et al., 2008; Förger et al., 2012) and none of the women in the included studies used tumor necrosis factor inhibitors (TNFi) or other biological DMARDs (bDMARDs) during pregnancy.

Nowadays, an increasing number of RA patients can reach remission or low disease activity thanks to the treat-to-target approach (T2T) with cDMARDs and bDMARDs. Being free of disease-related disability, young women living with RA can pursue their family plans and seek for a pregnancy. However, the management of treatment, especially bDMARDs, in relation to pregnancy has been debated in the last decade. The past general approach has been withdrawal of bDMARDs at positive pregnancy index, in order to avoid exposure during the early phases of pregnancy. As reassuring data about the use of bDMARDs during pregnancy accumulated, mostly about TNFi, recommendations from national and international societies have underlined their possible use during pregnancy, in the presence of a favorable benefit-risk ratio in the individual case (Flint et al., 2012; Gotestam Skorpen et al., 2016; Sammaritano et al., 2020).

The aim of this study was to assess the disease course of RA during pregnancy and pregnancy outcomes in relation to medication use and to identify possible risk factors for flares during pregnancy.

## Materials and Methods

### Patients

Data about RA pregnancies were retrospectively collected before conception and during each trimester and post-partum period. All the patients were prospectively followed at the multidisciplinary Pregnancy Clinic for Rheumatic Diseases at the University Hospital in Brescia between 2000 and 2018. Patients fulfilled the 2010 ACR/EULAR Classification Criteria for Rheumatoid Arthritis (Aletaha et al., 2010). All the patients signed a written informed consent. The study was approved the local Ethics Committee (Code N. 0025589—NP n. 1,647).

### Time points and clinical assessment

Data collection was performed at five time points: preconception visit (3–6 months before conception), during each trimester of pregnancy (first: 8–12 weeks of gestation, second: 18–24 weeks, third: 30–36 weeks), and up to 6 months after delivery.

The standard management consisted of a routine physical examination, assessment of diseases activity including the measurement of C-reactive protein (CRP) and recording of the current medication and complications. Presence of rheumatoid factor (RF), anti-citrullinated protein antibodies (ACPA), and bone erosions at X-rays were ascertained from the patients’ medical records.

### Assessment of disease activity and flare definition

Disease activity was assessed using the three-variable Disease Activity Score in 28 joints with CRP (DAS28-CRP3) since this score was shown to perform best in pregnancy (de Man et al., 2007). The mean disease activity scores were calculated at each time point. As previously described by *de Man et al.* (de Man et al., 2008), remission was defined as a DAS28-CRP < 2.6, according to the EULAR criteria, but using CRP instead of ESR. The proportions of women in clinical remission and with low, moderate, or high disease activity before pregnancy, during pregnancy, and after delivery were calculated.

As previously described by de Man et al. (de Man et al., 2008), flare was defined by an increase of DAS28-CRP3 between preconception visit and each time point >0.6 if the value was >3.2 or by an increase of DAS28-CRP3 >1.2 if the value was ≤3.2.

### Assessment of pregnancy outcome

Data on pregnancy outcome included early miscarriages (<10th gestational week), intrauterine foetal death (>10th gestational week), gestational age at delivery, mode of delivery, sex of the child, birth weight. Pregnancy complications were also recorded, including preterm deliveries (<37th gestational week), premature rupture of membranes (PROM), small for gestational age (SGA) babies (those with a birth weight below the 10th percentile for gestational age), and hypertensive disorders (gestational hypertension and pre-eclampsia). Data were retrieved from medical charts and by telephone interview when lacking. Mode of delivery was defined as spontaneous vaginal, induced vaginal, and caesarean section (elective or emergency).

### Statistical analysis

Continuous variables were reported as median and interquartile range (IQR), whereas categorical variables as proportion and/or percentage. Mann-Whitney test for continuous variables and Fisher’s exact test or Chi-square test for categorical variables were applied as appropriate. Logistic regression was applied for multivariate analysis. The model included those variables that had been associated with disease flare in the literature (e.g., negative prognostic factors such as ACPA or RF positivity) and variables related to drug exposure (e.g., stopping csDMARDs or bDMARDs) (see Table 2). *p*-values < 0.05 were considered as significant and Odds Ratio (OR) with 95% Confidence Interval (95% CI) was reported.

## Results

### Study cohort

A total of 83 pregnancies in 64 RA patients were identified. Eight (10%) pregnancies ended with early miscarriages (<10th gestational week), 1 (1%) with intrauterine foetal death (at 12th gestational week) and 1 (1%) with ectopic pregnancy.

The remaining 73 (88%) live-birth pregnancies in 63 patients (median age 35 years [IQR 30-38], median disease duration 68 months [IQR 30-159], positive ACPA 57%; positive RF 57%) were analysed. Eight women contributed with two live-birth pregnancies and one woman with three live-birth pregnancies.

Clinical, demographic, neonatal and breastfeeding features of 73 RA pregnancies are described in Table 1.

**TABLE 1:** Demographic, clinical, and neonatal characteristics of 73 pregnancies in patients with Rheumatoid Arthritis.

Age at conception (years), median (IQR)	35 (30-38)
Disease duration (months), median (IQR)	68 (30-159)
Positive RF	42 (57%)
Positive ACPA	42 (57%)
Bone Erosions	19/62 (30%)
History of > 1 cDMARD	20 (27%)
History of > 1 bDMARD	9 (12%)
Comorbidities on treatment	19 (26%)
Primigravida	26 (35%)
Preconception DAS28-CRP 3, median (min-max)	1.8 (1.1–4.1)
First trimester DAS28-CRP 3, median (min-max)	1.8 (1.1–5.9)
Second trimester DAS28-CRP 3, median (min-max)	1.9 (1.1–6.2)
Third trimester DAS28-CRP 3, median (min-max)	1.8 (1.1–5.2)
Post-partum period DAS28-CRP 3, median (min-max)	1.9 (1.1–6.2)
Gestational week at delivery, median (IQR)	39 (38–48)
Neonatal weight (grams), median (IQR)	3187 (2800–3500)
Breastfeeding	37 (50%)
Breastfeeding in LDA patients	23/37(62%)
Breastfeeding in no-LDA patients	12/21 (57%)
No breastfeeding due to maternal intake of non- compatible drugs	11 (15%)
No breastfeeding due to maternal choice	25 (35%)

Values indicate absolute numbers (percentage) unless otherwise stated.

ACPA, anti-citrullinated protein/peptide antibodies; CRP, C-reactive protein; DAS28-CRP3, disease activity score in 28 joints with CRP; DMARD, disease-modifying anti-rheumatic drug; bDMARD, biologic DMARD; cDMARD, conventional DMARD; GC, glucocorticoids; LDA, low disease activity; RA, rheumatoid arthritis; RF, rheumatoid factor.

### Disease course and medications

**FIGURE 1:**
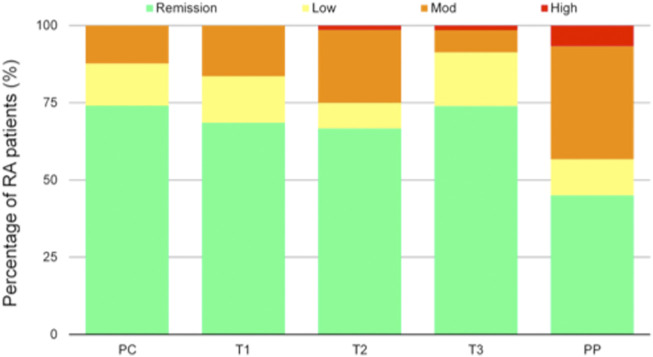
Disease activity according to Disease Activity Score in 28 joints (DAS28) during pregnancy and postpartum, classified as remission (DAS28 < 2.6), low disease activity (DAS28 2.6–3.2), moderate disease activity (DAS28 3.2–5.1), and high disease activity (DAS28 > 5.1). RA, rheumatoid arthritis; PC, preconception visit; T1 first trimester, T2 second trimester, T3 third trimester, PP post-partum.

Before conception, 54 (74%) patients were in remission, 10 (14%) had low disease activity and 9 (12%) moderate disease activity. None of the patients had high disease activity. During pregnancy, the percentage of patients with moderate disease activity increased during the first (12, 16.7%) and second trimester (17.2%). One patient (1.4%) had high disease activity during second and third trimester. After delivery, 27 (37%) patients were in remission, 7 (9.6%) had low disease activity, 22 (30.1%) moderate disease activity and 4 (5.5%) high disease activity (Figure 1). Twenty-four (40%) patients experienced a flare.

Table 2 reports on the use of drugs during each trimester and post-partum period (presented as overall use and start/resume of single drugs). Before conception, 41 (56%) patients were on treatment with cDMARDs and 30 (41%) on bDMARDs. Particularly, 23/29 (80%) patients who had stopped bDMARDs at positive pregnancy test resumed it during pregnancy (11/29, 38%) or after delivery (12/29, 41%) due to disease flare. Of note, 35 pregnancies (48%) were treated with low-dose acetylsalicylic acid and 8 (11%) with prophylactic dose heparin for obstetric indication and/or antiphospholipid antibodies positivity.

**TABLE 2:** Overall exposure to anti-rheumatic drugs and changes of treatment during pregnancy and post-partum period (numbers refer to patients on treatment at each time point).

	PC	T1		T2		T3		POST-PARTUM	
PDN, mg/day, median (IQR)	5 (3.5–5)	5 (3.5–6.25)		5 (3.5–6.25)		5 (3.5–7.5)		5 (2.8–6)	
		overall	start/resume	overall	start/resume	overall	start/resume	overall	start/resume
HCQ, n	26	36	10	38	2	35	0	34	9
SSZ, n	8	5	3	5	0	4	0	6	2
Cy-A, n	3	3	0	3	0	3	0	3	0
MTX/LEF, n	3	0	0	0	0	0	0	7	4
ETA, n	16	4	3	5	2	5	0	10	5
CTZ, n	5	1	1	4	3	5	1	6	1
ADA, n	4	0	0	1	1	1	0	3	2
GOL, n	3	0	0	0	0	0	0	1	1
Other bDMARDs, n	2	0	0	0	0	0	0	1	1

ADA, adalimumab; bDMARD, biological Disease-modifying anti-rheumatic drugs; Cy-A, cyclosporine; CTZ, certolizumab pegol; ETA, etanercept; GOL, golimumab; HCQ, hydroxychloroquine; IQR, interquartile range; LEF, leflunomide; MTX, methotrexate; PC, preconception visit; PDN, prednisone; SSZ, sulfasalazine; T1 first trimester, T2 second trimester, T3 third trimester.

No severe infections nor hospitalizations were observed. Women treated with bDMARDs during pregnancy and the post-partum period did not display a higher frequency of non-severe infections as compared to women treated with cDMARDs.

### Risk of flare

During pregnancy, flares occurred in 27 (37%) patients: 13 (18%) during first trimester, 10 (14%) during second trimester, and 6 (9%) during third trimester. Two patients experienced more than one flare. During the post-partum period, a flare occurred in 24 (40%) patients (median week after delivery 12, IQR 6-18). Two post-partum flares were observed also in 2 out of 10 (20%) women whose pregnancies had ended into spontaneous miscarriage.

By comparing pregnancies with and without flares (Table 3), flares during pregnancy were associated with elevated CRP and active disease in the first trimester, previous use of more than one bDMARDs, and the discontinuation of bDMARDs at positive pregnancy test. Active disease during first trimester was the only variable significantly associated with flare at the multivariate analysis (*p* 0.01, OR 5.4, CI 95% 1.48–19.55) (Table 3). Patients positive for RF and/or ACPA and patients with erosive disease did not display a higher frequency of flares as compared to patients without these features.

**TABLE 3:** Risk factors for disease flare during pregnancy.

	RA pregnancies (*n*=73)				
	Flare	No flare	OR	95%CI	p value
Positive RF	19/27 (70%)	25/46 (50%)	1.9	0.7–5.4	0.17
Positive ACPA	21/27 (77%)	24/46 (52%)	1.8	0.6–6.3	0.14
Erosive disease	9/27(33%)	10/46(21%)	1.5	0.7–7.2	0.27
History of > 1 cDMARD	13/27 (48%)	26/46 (56%)	0.7	0.3–1.8	0.48
History of >1 bDMARD	9/27 (33%)	5/46 (10%)	4.1	1.2–13.9	0.02
Elevated CRP before pregnancy	4/27 (14%)	7/46 (15%)	0.9	0.2–3.7	0.96
			6		
Active disease 1st trimester	9/27 (33%)	2/46 (4%)	11	2.16–55	0.01^§*^
Elevated CRP 1st trimester	12/25 (48%)	7/44 (16%)	4.8	1.6–15	0.04^§^
GC before pregnancy	19/27 (70%)	26/46 (56%)	1.8	0.6–5.2	0.24
cDMARD stopped before pregnancy	1/27 (3%)	0/46 (0%)	1.0	0.96–1.2	0.18
			3		
cDMARD stopped at positive pregnancy test	6/27 (22%)	10/46 (21%)	1.0	0.32–3.2	0.96
			2		
bDMARD stopped before pregnancy	0/27 (0%)	2/46 (4%)	0.9	0.9–1.02	0.27
			5		
bDMARD stopped at positive pregnancy test	15/27 (55%)	14/46 (30%)	2.8	1.06–7.06	0.03^§^

^*^p-value (multivariate analysis): (p 0.01. OR 5.4. CI 95% 1.48-19.55).

^§^Variables included in the multivariate analysis.

“Before pregnancy” refers to the period from 20 weeks prior to conception until the positive pregnancy test.

ACPA, anti-citrullinated protein/peptide antibodies; CRP, C-reactive protein; DMARD, disease-modifying anti-rheumatic drug; GC, glucocorticoids; RA, rheumatoid arthritis; RF, rheumatoid factor; OR, odds ratio.

### Pregnancy and neonatal outcome

Among 73 live-birth pregnancies, twenty-one (29%) had at least one complication. There were 8 preterm deliveries, of which 3 occurring <34th gestational week; 12 PROM, of which 8 preterm; 10 SGA newborns. One pregnancy was complicated by gestational hypertension and no case of pre-eclampsia was observed. By comparing pregnancies with and without the above-mentioned complications, no difference was observed in the history of adverse pregnancy outcomes in previous pregnancies, disease activity during pregnancy, values of CRP during pregnancy, glucocorticoids/cDMARDs/bDMARDs use during pregnancy.

Pregnancies ended with preterm delivery were characterised by higher values of CRP and DAS28-CRP3 in the first trimester as compared with pregnancies at term (10 mg/L (5–11) vs*.* 3 mg/L (2.5–5), *p* 0.01; 4.2 (1.9–4.5) vs*.* 1.9 (1.7–2.6), *p* 0.01, respectively). Preterm delivery was associated with the occurrence of flare (flare 27% vs*.* no-flare 7%, *p* 0.034; OR 4.625, 95% CI 1.03–20.83).

## Discussion

In the present study, we investigated the risk factors for disease flare during pregnancy in RA women who received preconception counselling and were mostly in good disease control at the time of conception (88% in remission or low disease activity at the time of conception, no patient with severely active disease). Flares during pregnancy and after delivery were observed in 37 and 40% of RA pregnancies, respectively. The occurrence of a flare during pregnancy was significantly associated with the withdrawal of bDMARDs (mostly TNFi) at positive pregnancy test. These results are concordant with other two studies. In 2015, Fischer-Betz et al. observed a flare in 16 (38%) pregnancies among 42 RA pregnancies. Women with RA who discontinued TNFi at conception displayed a high risk of flares during pregnancy, independently of known risk factors like RF and ACPA positivity and despite remission/low disease activity at conception (*p* 0.003 OR 8.2, 95% Cl 2.1–33.2) (Fischer-Betz et al., 2015). In 2017, *van den Brant et al.* observed disease flares in 29% of 75 pregnant RA women; the majority of flares occurred during the first trimester. Active disease and elevated CRP in early pregnancy along with the discontinuation of TNFi in the first trimester were identified as risk factors for flare (relative risk -RR 3.333, 95% CI 1.8–6.1, *p* 0.001) (van den Brant et al., 2017). More recently, *Förger et al.* showed that in RA patients with inactive disease, the discontinuation of TNFi before the 20th week of gestation did not result in active disease later in pregnancy as compared to continuing TNFi beyond the 20th week of gestation (Förger et al., 2019). However, it should be noted that patient-reported outcome measures were used to assess disease activity in this study and that the drugs were stopped later in gestation compared to the present study and to the above-mentioned studies (Fischer-Betz et al., 2015; van den Brandt et al., 2017).

The continuation of compatible drugs beyond conception and during pregnancy ensures a good control of maternal disease throughout pregnancy (Flint et al., 2012; Gotestam Skorpen et al., 2016; Sammaritano et al., 2020). As observed in this study, an active disease during the first trimester is a strong predictor of flare during pregnancy. On the other hand, low disease activity in the first trimester was shown to predict low disease activity or remission in the last trimester (Ince-Askan et al., 2017). Recent data from the PreCARA cohort showed that a modern treatment approach in pregnant RA patients, including T2T and the prescription of TNFi, yielded a low disease activity and remission during pregnancy, with 90.4% of patients achieving this target in the third trimester (Smeele et al., 2021).

In our study, flares occurred more frequently in patients previously treated with more than one bDMARDs. This finding suggests that patients with a more aggressive or difficult-to-treat disease have a higher risk of flaring up and need to continue compatible drugs beyond conception.

After delivery, women with RA are at risk of disease flare. Prospective studies before the year 2000 described high rates of postpartum disease worsening, ranging from 66 to 77% (Ostensen & Husby, 1983; Unger et al., 1983; Barrett et al., 1999). In a meta-analysis of five prospective studies from 2004 to 2013, a post-partum increase in disease activity was found in 46.7% of patients with RA (Jethwa et al., 2019). A recent study demonstrated that a tight control before pregnancy suppressed RA disease activity during pregnancy and in the postpartum period (Nakamura et al., 2021). In our cohort, 40% of patients experienced a flare. The progressive reduction of the rate of disease flares after delivery reflects a better management of RA over decades thanks to the use of csDMARDs and bDMARDs that can be continued during pregnancy and breastfeeding.

A good control of maternal disease activity is crucial not only to ensure a better RA course during pregnancy but also to favour a better pregnancy outcome. In this study, pregnancies that ended with a preterm delivery were characterised by higher values of CRP and DAS28-CRP3 in the first trimester as compared with pregnancies at term and they were associated with the occurrence of flare. These results are in agreement with a recent study conducted in 647 RA pregnant women between 2004 and 2017 (Smith et al., 2019). RA women had an increased risk of preterm deliveries versus the comparison group (RR 2.09, 95% CI 1.50–2.91), and an active disease at enrolment (aRR 1.58, 95% CI 1.10–2.27) and anytime during pregnancy (aRR 1.52, 95% CI 1.06–2.18) was associated with this complication (Smith et al., 2019). Another larger study carried on 440 RA pregnant women between 2005 and 2015 found that RA disease severity measured in early pregnancy was predictive of preterm delivery and SGA (Bharti et al., 2015), suggesting that tight control of disease activity in early pregnancy might improve birth outcomes. One retrospective study from 2014 showed no association between preterm deliveries and active disease at conception or throughout pregnancy (Langen et al., 2014). One might expect that the increased rate of preterm deliveries can be mediated through more glucocorticoid use to control disease flares, as this relationship has previously been documented in the literature (Smith et al., 2019). However, no association between preterm delivery and glucocorticoid use during pregnancy was observed in the present study. This finding could be accounted to the low dose of steroids (≤7.5 mg/day) used in our cohort (Table1). In our practice, pregnant women with active disease requiring dosages >7.5 mg were candidate to treatment with DMARDs, particularly bDMARDs, with the aim of minimizing the possible maternal and foetal adverse events linked to the continuous use median dosages of steroids during pregnancy. No association was also found between cDMARDs and/or bDMARDs use and adverse pregnancy outcomes, confirming recent data (Tsao et al., 2018a).

As a limitation of this study, we must mention the use of EULAR response criteria using the DAS-28 with CRP instead of ESR. In fact, as demonstrated by De Man et al. (De Man et al., 2009), disease activity can be measured the most reliably during pregnancy with the DAS28-CRP-3, because ESR increases physiologically during pregnancy. Another limitation of the present study was the inclusion of two or three pregnancies occurring in the same patient. The inclusion of a second or subsequent pregnancy might introduce bias, since it may represent a selection bias for women who previously had a good experience with their RA course during and after pregnancy and/or a good experience with the outcome of the pregnancy. On the other hand, it has been demonstrated that RA disease course in subsequent pregnancies cannot be predicted based upon previous pregnancies (Ince-Askan et al., 2016). We included pregnancies from 2000 to 2018, a long period in which the management of RA during pregnancy has been changing. The low number of pregnancies in each historical period did not allow us to make a sub-analysis upon calendar year.

The management of RA has improved in the past 2 decades with the introduction of a T2T approach and new and effective treatment options. This resulted in more women desiring pregnancy. As several rheumatology international guidelines for medication use in pregnancy and breastfeeding stated (Flint et al., 2012; Gotestam Skorpen et al., 2016; Sammaritano et al., 2020), there are multiple medications that are considered compatible with pregnancy and they should be continued during pregnancy if necessary. Despite the growing evidence about the safety of most of anti-rheumatic medications in pregnancy and breastfeeding, a frequent discontinuation of medications for RA, particularly in the first trimester, has been recently observed (Rebić et al., 2020) and women with RA resulted more than 3 times as likely to discontinue bDMARDs compared to those with inflammatory bowel disease (Tsao, et al., 2018b). This difference could be due to the old and widely held perception that RA spontaneously improves during pregnancy.

As we demonstrated in this study, a large proportion of RA women can experience a flare during pregnancy despite the good control of their disease activity before conception. Stopping bDMARDs early in pregnancy increases the risk of developing a flare during pregnancy. This information should be addressed during preconception counselling of women with RA, especially those with aggressive and/or refractory forms (e.g., history of more than one bDMARDs) and they should be offered to continue treatment during pregnancy to ensure control of maternal disease and prevent adverse pregnancy outcomes, particularly preterm delivery.

## Data Availability

The original contributions presented in the study are included in the article/supplementary material, further inquiries can be directed to the corresponding author.

## References

[B1] Aletaha D. Neogi T. Silman A. J. Funovits J. Felson D. T. Bingham C. O. (2010). 2010 rheumatoid arthritis classification criteria: An American college of rheumatology/European league against rheumatism collaborative initiative. Arthritis Rheum. 62 (9), 2569–2581. 10.1002/art.27584 20872595

[B2] Barrett J. H. Brennan P. Fiddler M. Silman A. J. (1999). Does rheumatoid arthritis remit during pregnancy and relapse postpartum? Results from a nationwide study in the United Kingdom performed prospectively from late pregnancy. Arthritis rheumatism 42 (6), 1219–1227. 10.1002/1529-0131(199906)42:6<1219::AID-ANR19>3.0.CO;2-G 10366115

[B3] Bharti B. Lee S. J. Lindsay S. P. Wingard D. L. Jones K. L. Lemus H. (2015). Disease severity and pregnancy outcomes in women with rheumatoid arthritis: Results from the organization of teratology information specialists autoimmune diseases in pregnancy project. J. Rheumatol. 42 (8), 1376–1382. 10.3899/jrheum.140583 25877497

[B4] de Man Y. A. Dolhain R. J. van de Geijn F. E. Willemsen S. P. Hazes J. M. (2008). Disease activity of rheumatoid arthritis during pregnancy: Results from a nationwide prospective study. Arthritis Rheum. 59 (9), 1241–1248. 10.1002/art.24003 18759316

[B5] de Man Y. A. Hazes J. M. van de Geijn F. E. Krommenhoek C. Dolhain R. J. (2007). Measuring disease activity and functionality during pregnancy in patients with rheumatoid arthritis. Arthritis Rheum. 57 (5), 716–722. 10.1002/art.22773 17530669

[B6] de Man Y. A. Hazes J. M. van der Heide H. Willemsen S. P. de Groot C. J. Steegers E. A. (2009). Association of higher rheumatoid arthritis disease activity during pregnancy with lower birth weight: Results of a national prospective study. Arthritis Rheum. 60 (11), 3196–3206. 10.1002/art.24914 19877045

[B7] Fischer-Betz R. Sander O. Specker C. Brinks R. Schneider M. (2015). High risk of flares during pregnancy in women with rheumatoid arthritis who discontinue treatment with TNF inhibitors at conception [abstract]. Arthritis Rheumatol. 67 (10). 10.1136/annrheumdis-2015-eular.4555

[B8] Flint J. Panchal S. Hurrell A. van de Venne M. Gayed M. Schreiber K. (2012). Pregnancy mediated improvement of rheumatoid arthritis. Swiss Med. Wkly. 142, w13644. 10.4414/smw.2012.13644 22811015

[B9] Förger F. Bandoli G. Luo Y. Robinson L. Johnson D. L. Chambers C. D. (2019). No association of discontinuing tumor necrosis factor inhibitors before gestational week twenty in well-controlled rheumatoid arthritis and juvenile idiopathic arthritis with a disease worsening in late pregnancy. Arthritis Rheumatol. 71 (6), 901–907. 10.1002/art.40821 30663847

[B10] Förger F. Vallbracht I. Helmke K. Villiger P. M. Østensen M. (2012). Pregnancy mediated improvement of rheumatoid arthritis. Swiss Med. Wkly. 142, w13644. 10.4414/smw.2012.13644 22811015

[B11] Götestam Skorpen C. Hoeltzenbein M. Tincani A. Fischer-Betz R. Elefant E. Chambers C. (2016). The EULAR points to consider for use of antirheumatic drugs before pregnancy, and during pregnancy and lactation. Ann. Rheum. Dis. 75 (5), 795–810. 10.1136/annrheumdis-2015-208840 26888948

[B12] Hazes J. M. Coulie P. G. Geenen V. Vermeire S. Carbonnel F. Louis E. (2011). Rheumatoid arthritis and pregnancy: Evolution of disease activity and pathophysiological considerations for drug use. Rheumatol. Oxf. Engl. 50 (11), 1955–1968. 10.1093/rheumatology/ker302 PMC3198908 21890617

[B13] Hench P. S. (1938). The ameliorating effect of pregnancy on chronic atrophic (infectious, rheumatoid) arthritis, fibrositis and intermittent hydrarthrosis. Proc. Staff Meet. Mayo Clin. 13, 161–167.

[B14] Ince-Askan H. Hazes J. Dolhain R. (2017). Identifying clinical factors associated with low disease activity and remission of rheumatoid arthritis during pregnancy. Arthritis Care Res. 69 (9), 1297–1303. 10.1002/acr.23143 27813290

[B15] Ince-Askan H. Hazes J. M. Dolhain R. J. (2016). Is disease activity in rheumatoid arthritis during pregnancy and after delivery predictive for disease activity in a subsequent pregnancy? J. Rheumatol. 43 (1), 22–25. 10.3899/jrheum.150565 26628599

[B16] Jethwa H. Lam S. Smith C. Giles I. (2019). Does rheumatoid arthritis really improve during pregnancy? A systematic review and metaanalysis. J. Rheumatol. 46 (3), 245–250. 10.3899/jrheum.180226 30385703

[B17] Langen E. S. Chakravarty E. F. Liaquat M. El-Sayed Y. Y. Druzin M. L. (2014). High rate of preterm birth in pregnancies complicated by rheumatoid arthritis. Am. J. Perinatol. 31 (1), 9–14. 10.1055/s-0033-1333666 23359233

[B18] Nakamura E. Kotani T. Hiramatsu Y. Hata K. Yoshikawa A. Matsumura Y. (2021). Simplified disease activity index and clinical disease activity index before and during pregnancy correlate with those at postpartum in patients with rheumatoid arthritis. Mod. Rheumatol. 31 (4), 809–816. 10.1080/14397595.2020.1829342 32990114

[B19] Ostensen M. Husby G. (1983). A prospective clinical study of the effect of pregnancy on rheumatoid arthritis and ankylosing spondylitis. Arthritis Rheum. 26 (9), 1155–1159. 10.1002/art.1780260915 6615567

[B20] Rebić N. Sayre E. C. Zusman E. Z. Amiri N. Baldwin C. De Vera M. A. (2020). Perinatal use and discontinuation of disease-modifying anti-rheumatic drugs and biologics in women with rheumatoid arthritis: A cohort study. Rheumatol. Oxf. Engl. 59 (7), 1514–1521. 10.1093/rheumatology/kez478 31628479

[B21] Sammaritano L. R. Bermas B. L. Chakravarty E. E. Chambers C. Clowse M. Lockshin M. D. (2020). 2020 American college of rheumatology guideline for the management of reproductive health in rheumatic and musculoskeletal diseases. Arthritis Rheumatol. 72 (4), 529–556. 10.1002/art.41191 32090480

[B22] Smeele H. T. Röder E. Wintjes H. M. Kranenburg-van Koppen L. J. Hazes J. M. Dolhain R. J. (2021). Modern treatment approach results in low disease activity in 90% of pregnant rheumatoid arthritis patients: The PreCARA study. Ann. Rheum. Dis. 80 (7), 859–864. 10.1136/annrheumdis-2020-219547 33568387 PMC8237196

[B23] Smith C. Förger F. Bandoli G. Chambers C. D. (2019). Factors associated with preterm delivery among women with rheumatoid arthritis and women with juvenile idiopathic arthritis. Arthritis Care Res. 71 (8), 1019–1027. 10.1002/acr.23730 PMC6384155 30133181

[B24] Tsao N. W. Lynd L. D. Sadatsafavi M. Hanley G. De Vera M. A. (2018b). Patterns of biologics utilization and discontinuation before and during pregnancy in women with autoimmune diseases: A population-based cohort study. Arthritis Care Res. 70 (7), 979–986. 10.1002/acr.23434 28973840

[B25] Tsao N. W. Sayre E. C. Hanley G. Sadatsafavi M. Lynd L. D. Marra C. A. (2018a). Risk of preterm delivery and small-for-gestational-age births in women with autoimmune disease using biologics before or during pregnancy: A population-based cohort study. Ann. Rheum. Dis. 77 (6), 869–874. 10.1136/annrheumdis-2018-213023 29496718

[B26] Unger A. Kay A. Griffin A. J. Panayi G. S. (1983). Disease activity and pregnancy associated alpha 2-glycoprotein in rheumatoid arthritis during pregnancy. Br. Med. J. 286 (6367), 750–752. 10.1136/bmj.286.6367.750 6402232 PMC1546996

[B27] van den Brandt S. Zbinden A. Baeten D. Villiger P. M. Østensen M. Förger F. (2017). Risk factors for flare and treatment of disease flares during pregnancy in rheumatoid arthritis and axial spondyloarthritis patients. Arthritis Res. Ther. 19 (1), 64. 10.1186/s13075-017-1269-1 28320445 PMC5359860

